# An Update on the Safety and Efficacy of Corneal Collagen Cross-Linking in Pediatric Keratoconus

**DOI:** 10.1155/2015/257927

**Published:** 2015-09-29

**Authors:** Hala El Rami, Elias Chelala, Ali Dirani, Ali Fadlallah, Henry Fakhoury, Carole Cherfan, George Cherfan, Elias Jarade

**Affiliations:** ^1^Faculty of Medicine, Saint-Joseph University, P.O. Box 11-5076, Riad El Solh, Beirut 1107 2180, Lebanon; ^2^Beirut Eye Specialist Hospital, P.O. Box 116-5311, Beirut, Lebanon; ^3^Mediclinic, Dubai Mall, Dubai, UAE

## Abstract

Keratoconus is a degenerative disease that affects adolescents and young adults and presents with variable thinning and conical deformation of the corneal apex. The resultant irregular astigmatism can progress to levels that can significantly affect everyday activities and overall quality of life. Therefore, stopping the progression of the disease is an essential part in managing patients with keratoconus. Corneal collagen cross-linking is a minimally invasive procedure that stiffens the anterior corneal stroma by creating strong covalent bonds between collagen fibrils. Over the past decade, many studies have proved its safety and efficacy in halting keratoconus progression in adults. This review of the literature highlights the growing trend towards using this treatment in pediatric keratoconic patients. In children, keratoconus tends to be more severe and fast progression is often encountered requiring closer follow-up intervals. Standard cross-linking shows comparable results in children with a good safety-efficacy profile during follow-up periods of up to three years. Further research is needed to standardize and evaluate transepithelial and accelerated cross-linking protocols as these could be of tremendous help in a population where cooperation and compliance are major issues.

## 1. Introduction

Keratoconus is a noninflammatory progressive degeneration of the cornea. It is characterized by bilateral often asymmetric thinning and conical protrusion of the corneal apex which account for irregular astigmatism and poor visual acuity [1, 2]. Its onset is classically around puberty, and progression is maximal during adolescence and early adulthood. When left untreated, spontaneous stabilization may occur during the third to fourth decade due to natural history of the disease, and stiffening of the corneal stroma by an increase in the number of cross-links between collagen fibrils may occur [3, 4]. Unfortunately, the progression of keratoconus for years before final stabilization may result in severe corneal scarring, and keratoplasty is the only treatment option left in up to 20% of patients [5, 6].

In an attempt to mimic the natural biomechanical stiffening that occurs with ageing, corneal collagen cross-linking with irradiated riboflavin was first introduced in the late 90s by Spörl et al. [7]. It induced the formation of strong covalent bonds between stromal collagen fibrils leading to a long-lasting increase in the biomechanical rigidity of the cornea [8, 9]. Nowadays, collagen cross-linking is an important asset in the management of keratoconus as a series of peer-reviewed studies have demonstrated its safety and efficacy in halting the progression of the disease and avoiding the need for corneal transplantation [10–15]. Some improvement in visual acuity, flattening of keratometric readings, and reduction in comatic aberrations have also been reported following cross-linking [12–15].

The encouraging safety-efficacy profile with follow-up ranging between 5 and 10 years in some series [13–15] has led many authors to consider cross-linking in pediatric patients and a rising number of reports on such attempts are being published. This review is intended to summarize the application and current status of corneal collagen cross-linking in pediatric keratoconus.

## 2. Epidemiology and Clinical Presentation of Keratoconus

The prevalence of keratoconus varies among different populations with an estimate of approximately 1/2000 individuals [1, 2].

It is often an isolated disease that is diagnosed in otherwise healthy subjects. However, systemic and/or ocular associations such as atopy, vernal keratoconjunctivitis, Down syndrome, retinitis pigmentosa, Leber congenital amaurosis, mitral valve prolapse, and noninflammatory connective tissue disorders such as Marfan and Ehlers-Danlos syndromes have been reported [1, 2, 16].

A genetic basis for keratoconus has been suspected and several candidate genes, including those coding for different types of collagen and proteinase inhibitors as well as antioxidant genes, have been investigated. Family history is classically found in up to 10% of patients [16–18]. This probably underestimates the true familial incidence of keratoconus as it does not take into account the subclinical disease known as “forme fruste” which corresponds to asymptomatic early stage keratoconus detected solely by means of modern corneal topography analysis and without evidence of progression over time [19]. When taking subclinical forms into account, first degree relatives have an estimated 15 to 67 times higher prevalence of keratoconus than that in the general population [20].

The disease usually starts at puberty. However, according to the CLEK study, the mean age at diagnosis is 27.3 ± 9.5 years and 90% of patients are diagnosed as early as 10 years of age or as late as 39 years of age [21]. This is due to the previously outlined highly variable expressivity of keratoconus with a small percent of patients (1%) having a “forme fruste” for a lifetime with good visual acuity and other patients progressing to stage 4 disease while still in their early twenties [22].

Clinical findings suggestive of keratoconus are as follows: progressive myopia and/or astigmatism with frequent change of glasses, suboptimal refraction suggesting the irregular nature of cylinder, scissoring of the red reflex, slit lamp examination showing thinning, apical protrusion, prominent corneal nerves, Vogt striae, Fleischer rings, and/or anterior stromal opacifications. Diagnosis is confirmed with corneal topography which typically shows the following: inferior to superior power asymmetry of more than 1.5 D, skewing of more than 20 degrees of the steepest radial axis above and below the horizontal meridian, and thin corneas with an inferotemporal displacement of “remarkable points,” such as the thinnest pachymetry, the steepest anterior, and the steepest posterior points. The definitive diagnosis will be based on a combination of clinical findings and topographic findings.

## 3. Main Characteristics of Keratoconus in the Pediatric Population

At presentation, the amount of visual impairment is variable and usually asymmetric in children with keratoconus. However, it rarely results in amblyopia because the development of visual function is completed at approximately 10 years of age. Many children have a history of eye rubbing and vernal keratoconjunctivitis.

At the time of diagnosis, the disease stage seems to be more advanced in younger patients. In a study of 476 eyes of 248 consecutive patients diagnosed with keratoconus, 17.2% were less than 20 years old and in this subgroup of patients, clinical findings were more severe [23]. In the study by Léoni-Mesplié et al., 27.8% of patients ≤15 years old had an Amsler-Krumeich stage 4 disease compared to 7.8% of patients ≥27 years old [24]. Deterioration in pediatric keratoconus occurs more frequently compared to adults with keratoconus. In a population of 1032 patients followed by the CLEK study group, progression occurred in 24% of cases and was maximal in patients less than 20 years old and minimal after 30 years of age [25]. Furthermore, recent studies have demonstrated more debilitating progression patterns with an increased likelihood of corneal opacities [26, 27] and keratoplasty [28, 29] in younger age patients.

## 4. Indications and Inclusion Criteria for Corneal Cross-Linking in Pediatric Keratoconus

In all published studies, emphasis was made about the documented progression of keratoconus prior to cross-linking. No clear definition for progression was found and each study defined its own progression criteria, summarized in Table 1. Progressive steepening, worsening asymmetry of keratometric readings, and decrease in central and/or thinnest pachymetry on consecutive measurements seem to be the most significant to consider when looking for deterioration as ametropia may change independently of keratoconus in children.

Keratoconus in patients less than 18 years old tends to be more aggressive and as previously outlined, severe deterioration is not exceptional. Therefore, rapid decision making is mandatory. Soeters et al. [38] diagnose progression in children within a 1 to 3 months' follow-up period versus 6 to 12 months' follow-up period in adolescents and adults while Chatzis and Hafezi [34] advise not to wait for progression to cross-link young corneas when the individual benefits outweigh the risks (i.e., severe disease in fellow eye, family history of progression to stage III-IV, family history of keratoplasty, etc.).

The major reported inclusion criteria for cross-linking in children with keratoconus were basically the same criteria reported for adults and included the following: thinnest pachymetry of more than 400 *μ*m [32–41], absence of corneal opacities [30, 32, 33, 35–43], no history of herpetic keratitis [32, 33, 36, 37, 39–41], absence of concurrent corneal infections [32, 33, 36, 37, 41], absence of severe dry eyes [32, 33, 36, 40, 41], absence of severe vernal keratoconjunctivitis [40], absence of concomitant autoimmune disease [32, 33, 36, 40], no history of previous ocular surgery [32, 33, 36, 38–41], and endothelial cell count of more than 1000 cells/mm^2^ [33].

## 5. Corneal Cross-Linking Techniques in Children

In the reported studies, all procedures were performed under topical anesthesia except in the study by Arora et al. [40] where general anesthesia was necessary in 8 out of 15 patients due to lack of cooperation. Premedication with 1 to 2% topical pilocarpine in the eye to be treated was often performed to induce a myotic pupil and protect the crystalline lens and posterior pole from light exposure.

The standard Dresden cross-linking protocol was carried out in most studies [31–36, 38, 40, 43] and consisted of the following:

1. Eight to 9 mm central epithelial debridement (Epi-Off technique) with a blunt metal spatula or a soft brush.
2. Photosensitization with an isotonic 0.1% Riboflavin mixed with 20% dextran solution, usually for 30 minutes before irradiation and then every 2 to 5 minutes during irradiation to maintain saturation of the cornea. In the study by Kodavoor et al. [43], hypotonic riboflavin was used in patients with thinnest pachymetry below 400 *μ*m, with the cutoff level being 350 *μ*m. In the study by Arora et al. [40], ultrasound pachymetry was repeatedly performed during the procedure, and hypotonic riboflavin was administered every 10 seconds during two minutes whenever pachymetry dropped below 400 *μ*m. This minimal corneal thickness has to be maintained throughout the procedure to avoid harmful endothelial side effects that would occur if oxygen radicals were created too deep (as in shallow corneas).
3. Uniform ultraviolet A irradiation at 3 mW/cm^2^ for 30 minutes, accounting for a surface dose of 5.4 J/cm^2^. The masking of the limbus and/or the treatment zone diameter were carefully selected to protect the limbal stem cells from the toxic effects of oxygen radicals generated by the procedure.

Other nonstandard techniques were also performed in children:

1. Transepithelial crosslinking (Epi-ON technique) [31, 36, 41, 42]: the epithelium was not removed and the riboflavin solution was supplemented with the epithelial penetration enhancers trishydroxymethyl aminomethane and sodium EDTA.
2. Accelerated cross-linking [37, 39]: a higher irradiance was delivered to reduce exposure time (i.e., 9 mW/cm^2^ for 10 minutes [37] or 30 mW/cm^2^ for 4 minutes [39] instead of 3 mW/cm^2^ for 30 minutes).

At the end of the cross-linking procedure, a bandage contact lens was applied and removed after complete epithelialization. A topical treatment with artificial tears, steroids, and antibiotics was prescribed.

## 6. Published Results of Corneal Cross-Linking in Pediatric Keratoconus

Studies' methodology, demographics, and overall safety-efficacy results are summarized in Table 2. Studies examining UVA cross-linking as a safe and effective modality in stabilizing keratoconus have been conducted in adults in the late nineties with preliminary results first published around 2003 [44]. Since then, numerous prospective trials have proved its long-term safety and efficacy in adults [10–15]. Data on cross-linking in children and young adolescents are scarce with only fourteen peer-reviewed studies reported so far [30–43]. This is explained by the fact that the studying of interventional procedures is more appropriate in a group of patients who are old enough to understand the nature of treatment and to get an informed consent.

### 6.1. Safety of Corneal Collagen Cross-Linking

Cross-linking showed a good safety profile in children, and no major complications have been reported with both standard and nonstandard techniques. Postoperative haze was often transient and resolved with no sequelae on topical steroids [32, 37, 40] except in the studies by Soeters et al. [38] and Kodavoor et al. [43] where significant haze occurred in 3.57% (2 out of 56 eyes) and 14.28% (5 out of 35 eyes) of patients, respectively. Delayed epithelialization of up to 10 days [34, 40] and transient glare and corneal edema [36] were also reported. There was no statistically significant loss in endothelial cell counts after cross-linking both in standard and accelerated techniques [32, 36, 37, 39, 41, 43]. However, longer follow-up periods are needed to make sure that the endothelial function does not suffer from any delayed side effects.

### 6.2. Efficacy of the Standard Cross-Linking Protocol

When performed with the standard protocol, cross-linking stabilized the disease process during the follow-up period which ranged between 12 and 36 months. When progression occurred despite cross-linking (one eye in the study by Chatzis and Hafezi [34] and 3 eyes in the study by Kodavoor et al. [43]), it was associated with persistent eye rubbing and/or vernal keratoconjunctivitis. Therefore, it is important to make sure that any underlying atopic disease that might contribute to later deterioration is well controlled at the time of cross-linking and the patient should be advised to stop rubbing his eyes. A redo cross-linking was performed by Chatzis and Hafezi [34] after 12 months of follow-up on the eye that showed progression. No significant complications occurred, the patient ceased eye rubbing, and he was stable at the 11 months' follow-up visit after redo cross-linking. Other reports have reported cases of adult patients who needed redo cross-linking [47, 48]. Risk factors associated with disease progression after cross-linking in these reports were the presence of neurodermatitis (associated with skin and eye rubbing) [47], female sex, and preoperative maximum *K*-readings superior to 58 D [48].

Improvement in uncorrected visual acuity (UCVA), best spectacle corrected visual acuity (BSCVA), and a significant flattening in *K*-readings were reported. Pachymetry remained stable. Soeters et al. [38] reported more corneal flattening and more visual acuity improvement in children compared to adults while Vinciguerra et al. [32] found better functional and morphological results in the subgroup of patients between 18 and 39 years old. However, in the study by Chatzis and Hafezi [34], improvement in *K*-readings occurred until the 24 months' follow-up visit with a regression to preoperative values at the 36 months' follow-up visit suggesting a possible decrease in the efficacy of cross-linking over time. This was not observed by Caporossi et al. [30] or Zotta et al. [33] who also reported 36 months' follow-up results. In adult populations, studies reported a sustainable effect over follow-up periods of up to ten years [15]. Knowing that natural cross-linking may occur with ageing of the corneal tissues [3, 4] and the possibility of spontaneous stabilization of keratoconus with advanced age, it is difficult to attribute the long-lasting stability observed in adults to either the initial surgery or the natural history of the disease. Furthermore, in the pediatric age group, corneal collagen remodeling and lay down of new collagen fibers occur at a higher rate than in adult corneas; hence, lay down of weak ectatic lamellae may exceed the capacity of the cross-linking process. Therefore, studies with longer follow-up periods are mandatory when cross-linking is performed at a very young age as cross-linking is more likely to have an expiry date with subsequent relapse.

### 6.3. Efficacy of the Nonstandard Cross-Linking Protocols

It is interesting to consider the nonstandard cross-linking techniques as these might solve some major cooperation issues that clinicians face when treating children. A significant decrease in the length of the treatment with an accelerated procedure or a “no-touch” reassuring transepithelial protocol would certainly make cross-linking easier to perform under topical anesthesia in the pediatric population. The accelerated cross-linking procedure showed an overall improvement in visual acuity and flattening of *K*-readings both in pediatric and adult populations [37, 39, 49, 50]. Although positive results are reported, it seems that the depth of treatment reached in accelerated cross-linking is less than the one achieved with the conventional technique with a mean demarcation line found at an average depth between 100 and 240 *μ*m versus 300 and 350 *μ*m with standard cross-linking [51, 52]. This finding might be related to the fact that the optimal irradiance and fluence time have not been clearly defined yet, and treatment settings were different among studies. On the other hand, while postoperative pain seems less when an Epi-ON procedure is performed [36, 41], controversy remains over the efficacy of transepithelial cross-linking. Traditionally, the epithelium is debrided prior to cross-linking because of the small and uneven penetration of riboflavin through the tight epithelial junctions resulting in insufficient stromal concentration. In transepithelial cross-linking, epithelial penetration enhancers such as benzalkonium chloride, tetracaine, surfactants, trishydroxymethyl aminomethane, and/or sodium EDTA were added to the riboflavin-dextran solution. The primary objectives behind keeping the corneal epithelium in the transepithelial protocol were the reduction of postoperative pain and infectious risk and the added corneal thickness of about 50 *μ*m, with possible reduction of phototoxicity and feasibility of cross-linking in thinner corneas. In the study by Magli et al. [36], transepithelial cross-linking was effective as no statistically significant difference was observed between standard and transepithelial techniques in terms of disease stabilization and improvement in children. The transepithelial procedure had fewer complications with less pain and no postoperative corneal edema. However, sample size was small (23 eyes in the transepithelial cross-linking group versus 16 eyes in the standard cross-linking group) and follow-up period was limited to only one year. Salman [41] found similar results in his small series of 22 eyes treated with transepithelial cross-linking and followed for a period of one year. Most authors reported progression of keratoconus in children and adults treated with the Epi-ON technique although transient improvement in keratoconus indices might occur [31, 42, 53]. Confocal microscopy performed on Epi-ON cross-linked corneas showed no significant modifications when compared to cross-linked corneas with standard or accelerated Epi-OFF protocols [51]. Some corneal stiffening was demonstrated in the study by Scarcelli et al. [54] on porcine corneas but it was 70% less pronounced than in Epi-OFF standard technique. In his study, corneal stiffening was strongly related to irradiance time suggesting that optimizing the light dose might increase the efficacy of the transepithelial protocol in future studies.

## 7. Conclusion

Keratoconus in children tends to be more severe than in adults and rapid deterioration can occur requiring more frequent follow-up intervals. Moreover, the decision to cross-link pediatric corneas should be prompt as studies have proved the safety and efficacy of the standard cross-linking protocol in halting disease progression. However, studies with larger samples and longer follow-up need to be performed to determine if stabilization is long-lasting in children. When dealing with patients younger than 14 years of age, compliance is a major issue making nonstandard accelerated or transepithelial protocols appeal to clinicians. More research is needed to determine and standardize the most effective treatment parameters for these protocols and randomized controlled trials would prove essential in comparing these techniques to the standard cross-linking protocol in terms of long-lasting efficacy.

## Figures and Tables

**Table 1:** Variability of the criteria used to define keratoconus progression in different studies.

Study	Visual acuity	Refraction	Keratometry	Pachymetry	Topography	Considerations
Caporossi et al. [30]	UCVA/BSCVA decrease ≥ 1 Snellen line	ΔSph or ΔCyl > 0.5 D	Δ*K*_mean_ > 0.5 D	Decrease in thinnest pachymetry ≥ 10 *μ*m	ΔSAI/SI > 0.5 D	At least 2 parameters in 3 months

Caporossi et al. [31]	UCVA/BSCVA decrease ≥ 1 Snellen line	ΔSph or ΔCyl > 0.5 D	Δ*K*_max⁡_ > 1 D	Decrease in thinnest pachymetry ≥ 10 *μ*m	ΔSAI/SI > 0.5 D	At least 3 parameters (1 clinical and 2 instrumental) in 3 months

Vinciguerra et al. [32]		Change in Sph or Cyl ≥ 3 D	Δ*K*_mean_ ≥ 1.5 D on 2 consecutive topographies	Decrease in CCT ≥ 5% on 3 consecutive tomographies		Any parameter in 3 months

Zotta et al. [33]		ΔSE > 0.75 D	Δ*K*_max⁡_ of cone apex > 0.75 D			Any parameter in 6 months

Chatzis and Hafezi [34]			Δ*K*_max⁡_ > 1 D			Follow-up period of maximum 12 months

Bakshi et al. [35]		ΔCyl ≥ 1.5 D	Δ*K*_max⁡_ ≥ 1.5 D			Any parameter at 3 time points in 12 months

Magli et al. [36]		ΔCyl > 1 D	Δ*K*_max⁡_ of cone apex > 1 D			Any parameter in 6 months

Shetty et al. [32, 37]			Δ*K*_max⁡_ > 1–1.5 D with corresponding change in refraction	Decrease in thinnest pachymetry ≥ 5%		Any parameter in 6 months

Zotta et al. [33]		ΔCyl ≥ 1 D and ΔSE ≥ 0.5 D	Δ*K*_max⁡_ ≥ 1 D			All 3 parameters on consecutive examinations

UCVA: uncorrected visual acuity, BSCVA: best spectacle corrected visual acuity, Sph: sphere, Cyl: cylinder, SAI: surface asymmetry index, SI: symmetry index, CCT: central corneal thickness, SE: spherical equivalent, and Δ: increase in.

**Table 2:** Cross-linking in pediatric keratoconus: summary of the studies' characteristics and overall results.

Study	Type	*N* patients/eyes	Age (yrs.)	M/F ratio	Amsler-Krumeich classification	F-up before CXL (mo.)	CXL Technique	F-up after CXL (mo.)	Safety	Efficacy
Caporossi et al. [30]	P	152/77	10–18	4/1	≤III	12–36	Standard	36	+	+

Caporossi et al. [31]	P	10/10	11–18				Transepithelial	24	+	−

Vinciguerra et al. [32]	P	40/40	9–18	31/9	II	3	Standard	24	+	+

Buzzonetti and Petrocelli [42]	P	13/13	8–18	12/1	≤III		Transepithelial	18	+	−

Zotta et al. [33]	R	4/8	11–15	1/3		6–9	Standard	36	+	+

Chatzis and Hafezi [34]	R	42/59	9–19	29/13	I–IV		Standard	36	+	−

Arora et al. [40]	P	15/15	10–15		I-II		Standard	12	+	+

Bakshi et al. [35]	R	9/9	11–17	1/0		12	Standard	24	+	+

Magli et al. [36]	R	19/23 11/16	12–17 12–17	14/5 8/3		6 6	Standard Transepithelial	12	+	+

Shetty et al. [37]	P	18/30	11–14				Accelerated 9 mW/cm^2^ for 10 minutes	24	+	+

Ozgurhan et al. [39]	R	38/44	9–18	14/5			Accelerated 30 mW/cm^2^ for 4 minutes	24	+	+

Salman [41]	P	22/22	13–18	9/2		12	Transepithelial	12	+	+

Soeters et al. [38]	P	—/31	12–17				Standard	12	+	+

Kodavoor et al. [43]	R	24/35	9–16				Standard	12	+	+

Viswanathan et al. [45]	P	18/25	8–17	13/5	I–III		Standard	6–48	+	+

Sabti et al. [46]	C	1/2	4	1/0		3	Standard	36	+	+

F-up: follow-up, CXL: cross-linking, P: prospective, R: retrospective, C: case report, (+): favorable outcome, and (−): unfavorable outcome.
